# Mechanical and degradation properties of advanced platelet-rich fibrin (A-PRF), concentrated growth factors (CGF), and platelet-poor plasma-derived fibrin (PPTF)

**DOI:** 10.1186/s40729-017-0081-7

**Published:** 2017-05-02

**Authors:** Kazushige Isobe, Taisuke Watanebe, Hideo Kawabata, Yutaka Kitamura, Toshimitsu Okudera, Hajime Okudera, Kohya Uematsu, Kazuhiro Okuda, Koh Nakata, Takaaki Tanaka, Tomoyuki Kawase

**Affiliations:** 1 Tokyo Plastic Dental Society, Kita-ku, Tokyo, Japan; 2 Division of Dental Implantology, Niigata University Medical and Dental Hospital, Niigata, Japan; 3 Division of Periodontology, Institute of Medicine and Dentistry, Niigata University, Niigata, Japan; 4 Bioscience Medical Research Center, Niigata University Medical and Dental Hospital, Niigata, Japan; 5 Department of Materials Science and Technology, Niigata University, Niigata, Japan; 6 Division of Oral Bioengineering, Institute of Medicine and Dentistry, Niigata University, Niigata, Japan

**Keywords:** Platelet-rich fibrin, Concentrated growth factors, Platelet-poor plasma, Young’s modulus, Fibrin fiber, Degradability

## Abstract

**Background:**

Fibrin clot membranes prepared from advanced platelet-rich fibrin (A-PRF) or concentrated growth factors (CGF), despite their relatively rapid biodegradability, have been used as bioactive barrier membranes for alveolar bone tissue regeneration. As the membranes degrade, it is thought that the growth factors are gradually released. However, the mechanical and degradable properties of these membranes have not well been characterized. The purpose of this study was to mechanically and chemically characterize these membranes.

**Methods:**

A-PRF and CGF clots were prepared from blood samples collected from non-smoking, healthy donors and were compressed to form 1-mm-thick membranes. Platelet-poor plasma-derived fibrin (PPTF) clots were prepared by adding bovine thrombin to platelet-poor plasma. A tensile test was performed at the speed of 1 mm/min. Morphology of the fibrin fibers was examined by SEM. A digestion test was performed in PBS containing trypsin and EDTA.

**Results:**

In the tensile test, statistical difference was not observed in Young’s modulus, strain at break, or maximum stress between A-PRF and CGF. In strain at break, PPTF was significantly weaker than CGF. Likewise, fibrin fiber thickness and crosslink density of PPTF were less than those of other membranes, and PPTF degraded faster than others.

**Conclusions:**

Although the centrifugal conditions are different, A-PRF and CGF are prepared by essentially identical mechanisms. Therefore, it is conceivable that both membranes have similar mechanical and chemical properties. Only PPTF, which was prepared by a different mechanism, was characterized as mechanically weaker and enzymatically more degradable.

## Background

Platelet-rich fibrin (PRF), a self-clotted preparation of platelet-concentrated, blood-derived biomaterials, is prepared solely by contact activation of intrinsic coagulation pathways through centrifugation without addition of coagulation factors [1, 2]. Therefore, the preparation protocol is drastically simplified, and the resulting clot can be handled easily with forceps. PRF is further modified to two types: A-PRF, an advanced type that is expected to contain greater numbers of white blood cells [3] and concentrated growth factors (CGF), which is prepared under a facilitated intrinsic coagulation cascade [4]. Since these preparation protocols are similar and share the same principle of clot formation, A-PRF and CGF clots are not easy to differentiate either macroscopically or microscopically.

In clinical settings, both A-PRF and CGF preparations have been applied as barrier membranes and/or as carriers of growth factors to facilitate wound healing and tissue regeneration. However, their mechanical properties as barrier membranes have not been investigated sufficiently. For example, there is no available evidence as to which membrane is mechanically tougher. In addition, because the fibrin membranes degrade gradually at the implantation site in vivo, it is poorly understood how their mechanical properties change during the degradation process.

Degradability is also closely related to growth factor release, a phenomenon that is a key parameter in the efficacy at the implantation site. Recently, it has been demonstrated that growth factors are concentrated in A-PRF/CGF clots and released with time [5–10]. These experimental systems simulated the initial phase of growth factor release by simple diffusion; however, the simulation experiments were performed using conventional culture media in the absence of serum or proteases, which is not an appropriate simulation system of in vivo conditions. Therefore, it is apparent that growth factor release by degradation of fibrin fibers [11] is not well simulated. In the data obtained from our previous [12] and preliminary studies, fibrin clots can be maintained without substantial degradation under similar protease-free conditions for longer than a week. However, clinicians have frequently claimed based on their clinical experiences that fibrin clots applied to surgical sites, e.g., socket after tooth extraction, are almost completely degraded within a week or two. This observation is supported by several clinical review articles [13, 14].

In this study, we hypothesized that the mechanical properties of the fibrin membrane are closely related to its degradability. We compared these characteristics among A-PRF, CGF, and PPTF membranes through tensile and digestion tests.

## Methods

### Preparation of A-PRF and CGF clots

Blood samples were collected from four non-smoking, healthy, male volunteers with ages ranging from 27 to 56 years. Although having lifestyle-related diseases and receiving medication, these donors had no hindrance in daily life. The study design and consent forms for all procedures performed with the study subjects were approved by the ethical committee for human subjects at Niigata University School of Medicine in accordance with the Helsinki Declaration of 1975 as revised in 2008.

As described previously [6, 15, 16], blood samples (~9.0 mL) collected without anticoagulants using vacuum plain glass tubes (A-PRF+; Jiangxi Fenglin Medical Technology Co. Ltd., Fengcheng, China) or conventional vacuum plain glass tube (Plain BD Vacutainer Tube; Becton, Dickinson and Company, Franklin Lakes, NJ, USA) from the same donors were immediately centrifuged by an A-PRF centrifugation system (A-PRF12; DRAGON LABORATORY Instruments Ltd., Beijing, China) or a Medifuge centrifugation system (Silfradent S. r. l., Santa Sofia, Italy). After eliminating the red blood cell (RBC) fractions, the resulting A-PRF and CGF clots were compressed using a stainless-steel compression device and preserved between wet gauze until mechanical testing (usually for a maximum of 3 h).

### Preparation of PPP clots

To prepare platelet-poor plasma (PPP), peripheral blood (~9.0 mL) was collected using syringes containing A-formulation of acid-citrate-dextrose (ACD-A) (1.0 mL; Terumo, Tokyo, Japan) and immediately fractionated by the conventional double-spin method [17, 18]. The supernatant was collected as the PPP fraction. To prepare fibrin clots, bovine thrombin (Liquid Thrombin MOCHIDA Softbottle, Mochida Pharmaceutical Co. Ltd., Tokyo, Japan) was added to the PPP at a final volume percentage of 2.5% (v/v) at ambient temperature in glass chambers. The resulting PPP clots, which is designated as platelet-poor, thrombin-activated fibrin (PPTF), were compressed and preserved between wet gauze until mechanical testing (usually for a maximum of 3 h).

### Determination of water content in fibrin clots

After excess amounts of exudate were quickly absorbed by the dry gauze, wet weights of freshly prepared A-PRF, CGF, and PPTF clots were measured using an electric balance. After compression with the stainless compressor, their weights were measured again. The compressed clots were then dried by heating at 140 °C for 30 min and were weighed in a pre-heated moisture analyzer (MA35; Sartorius Corporate Administration GmbH, Goettingen, Germany).

### Mechanical testing

The mechanical properties of gel sheets were measured at a stretching speed of 1 mm/min with a desktop universal testing machine (EZ test; Shimadzu, Kyoto, Japan), of which maximum load cell capacity was 500 N under standard ambient conditions at 25 ± 3 °C and 50 ± 25% RH. The samples were gripped by clamps at each end (using slip-proof rubber sheets to prevent slippage) such that the initial apparent gauge length (the distance between clamp faces) was set to 10 mm for all the samples tested.

Young’s modulus, maximum tensile strength, and tensile strain at break were obtained from the stress-strain plot. Stress was calculated by dividing the force by the initial tissue cross-sectional area, assuming a rectangular geometry (Table 1). The modulus for each sample was determined from the slope of the stress-strain curve during the apparent strain of 50–150% where the curve was almost linear while the sample had a sag during the apparent strain of 0–50%. The strain was recalculated to eliminate the sag when the Young’s modulus and the maximum strain at break.

**Table 1:** Similarity in size and stretching property of A-PRF and CGF membranes

	Size (W × L mm)	Stretching (times longer)	Number
A-PRF	8.6 ± 1.2 × 27.5 ± 3.5	2–4	9
CGF	8.4 ± 0.8 × 27.6 ± 2.5	2–4	9
PPTF	8.3 ± 1.2 × 31.8 ± 2.1	2–4	3

According to the definition in the Handbook of Polymer Testing [19], “Young’s modulus” is the modulus of elasticity in tension and defined as ratio of stress difference to the corresponding strain difference (stress/strain). In this study, the initial elongation property (slope) was evaluated to determine Young’s modulus. “tensile strain at break” is defined as tensile strain at the tensile stress at break, if it breaks without yielding. “Maximum tensile stress” sustained by the test specimen during a tensile test represents tensile strength.

### Digestion test

A-PRF/CGF/PPTF clots (1 mm thick) were compressed in the stainless-steel compressor [16] and were punched out (φ8 mm) using a biopsy punch (Kai Corp., Tokyo, Japan). After repeatedly rinsing the disks with PBS to eliminate as much serum as possible, the disks were immersed into 4 mL of 0.05% trypsin plus 0.53 mM EDTA (Invitrogen, Carlsbad, CA, USA) in a 35-mm dish inside a CO_2_ incubator. Fibrin is well known to be specifically degraded by plasmin in vivo; however, because it takes a long time to determine degradation using plasmin in vitro [12] and because fibrin could be degraded also by other proteases in vivo, we used trypsin plus EDTA, which is usually used in cell culture, in this study.

After pipetting the digestion solution, 50 μL of the digestion solution was collected every 20 min and was stored at −20 °C until protein measurement. Protein levels, which can be considered primarily as levels of digested fibrin fiber, were then determined by a BCA protein assay kit (Takara Bio, Kusatsu, Japan). The protein levels at the time point when the initial fibrin disks were completely digested overnight were evaluated at 100%.

### Scanning electron microscopy (SEM)

The PRF clots that were compressed in a stainless-steel compressor, were fixed with 2.5% neutralized glutaraldehyde, dehydrated with a series of ethanol solutions and *t*-butanol, freeze-dried, and then were examined under a scanning electron microscope (TM-1000; Hitachi, Tokyo, Japan) with an accelerating voltage of 15 kV, as described previously [16].

### Statistical analysis

The data were expressed as mean ± standard deviation (SD). For multi-group comparisons, statistical analyses were conducted to compare the mean values by one-way analysis of variance (ANOVA) followed by Dunn’s multiple-comparison test (SigmaPlot 12.5; Systat Software, Inc., San Jose, CA, USA). Differences with *P* values < 0.05 were considered significant.

## Results

The main purpose of this study was to compare A-PRF with CGF preparations to find possible differences in mechanical properties. As shown in Table 1, the sizes of A-PRF clots compressed to membranes were 8.6 ± 1.2 mm (W) × 27.5 ± 3.5 mm (L) and very similar to those of CGF clots (8.4 ± 0.8 mm × 27.6 ± 2.5 mm). As reference, PPTF membranes were also prepared by adding CaCl_2_ to liquid PPP preparations using a molding glass chamber. The size of PPP membranes prepared by adding thrombin, designated PPTF in this study, was 8.3 ± 1.2 mm × 31.8 ± 2.1 mm. Furthermore, when subjected to the tensile test, both membranes could be stretched two to four times their original length. As shown in Table 2, the water content of A-PRF clots was very similar to that of CGF clots. However, PPTF clots contained significantly less amounts of water than both A-PRF and CGF clots.

**Table 2:** Comparison of water content of A-PRF, CGF, and PPTF clots

	Wet weight (g)	Dry weight (g)	Water content (%)
A-PRF	1.905 ± 0.416	0.043 ± 0.014*	97.8 ± 0.7*
CGF	1.753 ± 0.302	0.035 ± 0.009*	98.0 ± 0.6*
PPTF	1.774 ± 0.287	0.066 ± 0.004	96.2 ± 0.7

*N* = 5

*P < 0.05 compared with PPTF

**Fig. 1:**
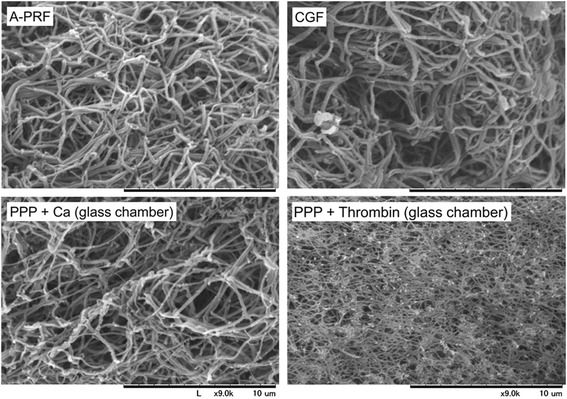
Surface microstructures of A-PRF, CGF, and fibrin clots prepared by PPP + CaCl_2_ and PPTF (fibrin clots prepared by PPP and thrombin). Similar observations were obtained from other three independent blood samples. *Scale bar* = 10 μm. *Note*: the same magnification (×9000) was used in all the SEM images shown here

Surface microstructures of various fibrin clots, including A-PRF and CGF clots, were compared, as shown in Fig. 1. Based on SEM examinations, CGF clots contained thicker fibrin fibers than A-PRF clots. PPP clots prepared by adding CaCl_2_ were composed mainly of relatively thin fibers. In contrast, PPTF clots were easily distinguishable from the other three clot types and were composed of highly crosslinked fibers that were the thinnest observed.

**Fig. 2:**
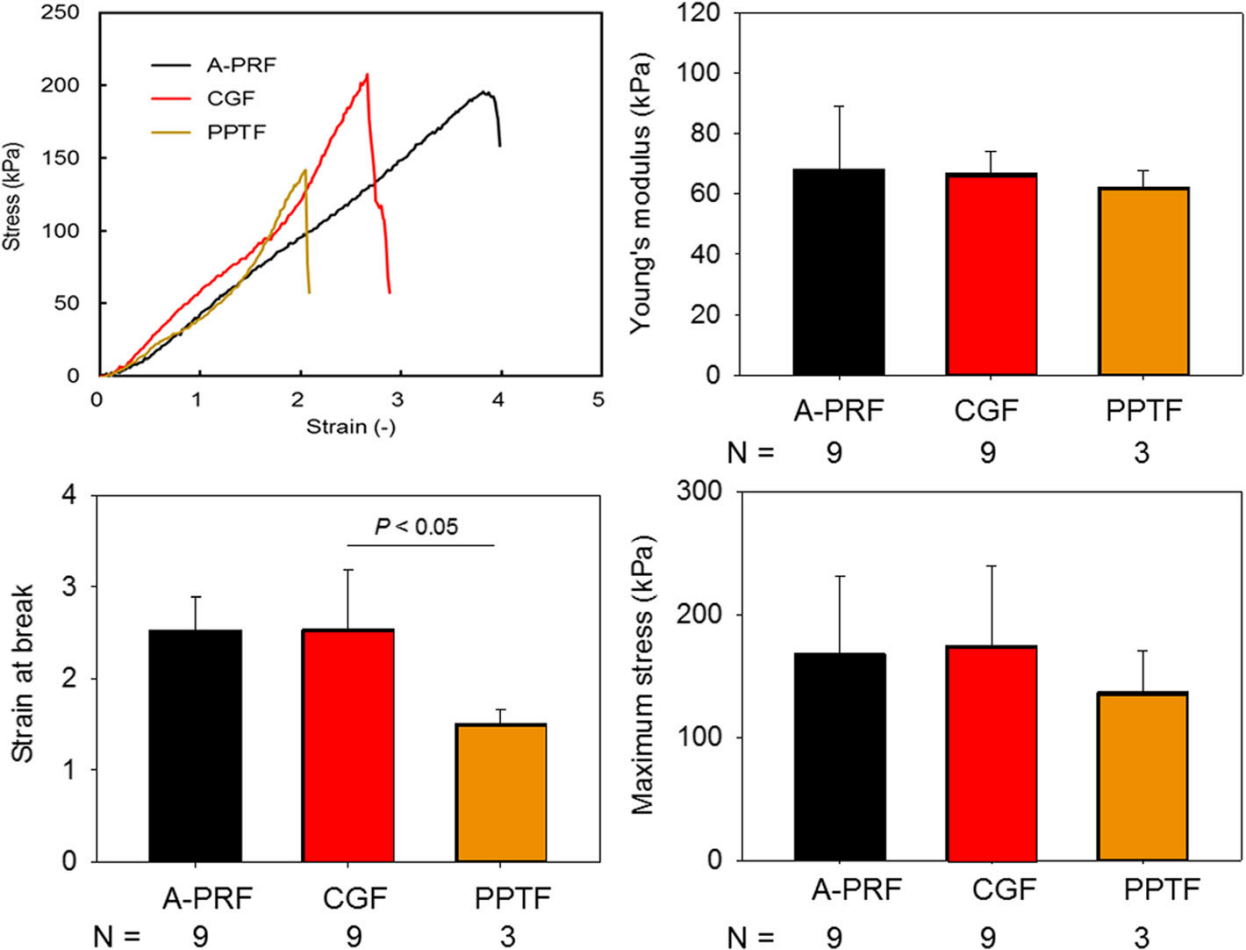
Representative stress-strain curves for A-PRF and CGF membranes and mechanical properties (Young’s modulus, strain at break, and maximum stress) of A-PRF, CGF, and PPTF membranes. *N* = 3–9

Individual membrane types were examined by a tensile test and were characterized by three parameters: (1) Young’s modulus, (2) strain at break, and (3) maximum stress in the stress-strain curves. As shown in Fig. 2, no significant differences in both Young’s modulus and maximum stress were observed among A-PRF, CGF, and PPTF membranes. However, in strain at break, PPTF membranes were significantly inferior to CGF membranes.

**Fig. 3:**
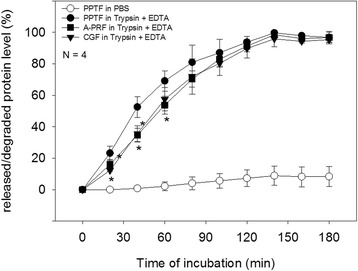
Enzymatic degradability of A-PRF, CGF, and PPTF membranes. Each membrane disk (φ8 mm, 1 mm thick) was immersed in PBS containing trypsin and incubated in a CO_2_ incubator. *N* = 4. The *asterisks* represent significant differences (*P* < 0.05) compared with A-PRF at the same time points

Degradability of individual membrane types was examined in PBS containing trypsin and EDTA. As shown in Fig. 3, PPTF membranes degraded significantly faster than A-PRF and CGF membranes. This disparity in degradability was observed at 20 and 40 min.

## Discussion

In this study, we found no apparent differences between A-PRF and CGF clot microstructures, especially in fibrin fiber thickness or crosslink density. However, in PPTF clots, which were prepared through direct conversion of fibrinogen by thrombin, fibrin fiber thickness and their crosslink density were substantially thinner and higher, respectively, than those of either A-PTF or CGF clots. This finding was supported by the water content data, which revealed that significantly less amounts of water were contained in PPTF clots. These data are summarized along with the centrifugal conditions in Table 3.

**Table 3:** Summaries of preparation procedures, relative mechanical, degradation, and related properties of A-PRF, CGF and PPTF

		A-PRF	CGF	PPTF
Centrifugal conditions		198 *g* × 8 min	692 *g* × 2 min^a^ 547 *g* × 4 min 692 *g* × 4 min 855 *g* × 3 min	580 *g* × 8 min (1st)^b^ 1060 *g* × 8 min (2nd)
Anticoagulants		None	None	ACD-A
Coagulation factors		None	None	Thrombin
Mechanical strength		Tough	Tough	Moderate
Serum retention		High	High	Medium
Degradation		Moderate	Moderate	Fast
Fibrin fibers	Thickness	Thick	Thick	Thin
	Crosslink density	Low	Low	High

^a^The centrifugal force was automatically changed by the specific program of centrifuge

^b^PPP was prepared by the double-spin method

Since the ratio of surface area to volume is known to be a significant factor for degradation of polymer material [20], these structural characteristics can be correlated to their degradability. As expected, we demonstrated that PPTF membranes degraded faster than other self-clotted fibrin membranes and A-PRF and CGF degradation rates were almost identical. However, it has not yet been clarified if those structural characteristics are correlated to mechanical properties.

In the tensile test, we again found no significant difference in any parameters evaluated among A-PRF, CGF, and PPTF membranes. However, in the strain at break, PPTF membranes were broken by a significantly weaker tensile force. The order of this parameter from high to low was CGF ≈ A-PRF > PPTF. As described above, the order of degradability was PPTF > CGF ≈ A-PRF, which is the reverse of the mechanical strength. Despite higher crosslink density, fibrin fibers formed in PPTF clots were substantially thinner and therefore they are probably not capable of bearing higher tensile forces. The manufacturer explains that the difference between PRF and CGF is related to the centrifugation techniques; programmed switching between acceleration and deceleration facilitates both conversion of fibrinogen to fibrin and their polymerization more efficiently than centrifugation at fixed speeds. However, as far as we examined, CGF is identical to A-PRF in terms of mechanical and degradable properties.

Growth factor release is a key function of these fibrin clots for tissue regeneration. Our previous study [16] demonstrated that CGF membranes compressed by the stainless steel compression device contain significantly higher levels of growth factors even after releasing approximately 85% of exudate. Repeated rinsing with PBS failed to completely remove the growth factors from CGF membranes. The rinsed CGF membranes retained angiogenic effects in ex vivo and in vitro experimental systems. Considered together, these data imply that significant amounts of the growth factors are secured in CGF membranes, specifically in fibrin fibers. Similar functions were found in A-PRF and PPTF membranes. Therefore, it is thought that two distinct mechanisms are involved in controlled release of growth factors in exudate-depleted fibrin membranes: growth factors adsorbed to fibrin fibers and growth factors caged in platelets aggregated on fibrin fibers.

The initial phase of growth factor release from fibrin clots is mainly attributed to simple diffusion. In contrast, the late phase, i.e., the delayed growth factor release, is probably due to degradation of fibrin fibers and platelet membranes. We think that these combined releasing mechanisms determine how long the individual fibrin clot types last for tissue regeneration. This complex process of growth factor release from PRF (CGF) membranes should be investigated more carefully by developing appropriate experimental conditions.

## Conclusions

In the mechanical parameters and degradability we tested, CGF membranes were almost identical to A-PRF membranes. In contrast, PPTF membranes were mechanically weaker and highly degradable. Therefore, we conclude that all of these fibrin membranes are tough enough to serve as barrier membranes; however, we should pay attention to their degradability and choose an appropriate membrane type depending on the purpose of treatment and the condition of wounds or bone defects.

## Abbreviations

ACD: Acid citrate dextrose solution
A-PRF: Advanced platelet-rich fibrin
CGF: Concentrated growth factors
PPTF: Platelet-poor plasma-derived, thrombin-activated fibrin
PRP: Platelet-rich plasma
